# Visual impairment and its predictors among people living with type 2 diabetes mellitus at Dessie town hospitals, Northeast Ethiopia: institution-based cross-sectional study

**DOI:** 10.1186/s12886-022-02292-3

**Published:** 2022-02-03

**Authors:** Mohammed Abdu Seid, Adugnaw Ambelu, Mengistie Diress, Yigizie Yeshaw, Yonas Akalu, Baye Dagnew

**Affiliations:** 1 Unit of Human Physiology, Department of Biomedical Sciences, College of Health Sciences, Debre Tabor University, P. O. Box 272, Debre Tabor, Ethiopia; 2 Department of Human Physiology, College of Medicine and Health Sciences, University of Gondar, P. O. Box 196, Gondar, Ethiopia

**Keywords:** Visual impairment, Type 2 diabetes mellitus, Ethiopia

## Abstract

**Background:**

Visual impairment is a functional limitation of the eye(s) that results in reduced visual acuity, visual field loss, visual distortion, perceptual difficulties, or any combination of the above. Type 2 diabetes mellitus is one of the common causes of visual impairment. Since there is no study conducted in Ethiopia so far in this regard, the current study aimed to determine the prevalence and predictors of visual impairment among people living with diabetes at Dessie town Hospitals, Northeast Ethiopia.

**Methods:**

Institution based cross-sectional study was carried out from 15 February to 15 March 2020 using simple random sampling to recruit study participants among type 2 diabetes. Visual impairment was measured using visual acuity test. We used Epi Data 3.1 and SPSS version 22 for data entry and statistical analysis, respectively. Bi-variable binary logistic regression was performed to check independent association of each factor with visual impairment. After selecting candidate variables at *p* < 0.25, we computed multivariable binary logistic regression to identify statistically associated factors of visual impairment. The degree of association was determined using adjusted odds ratio with 95%CI. In the final model, statistical significance was declared at *p* < 0.05.

**Results:**

Three hundred and twenty-two people living with T2DM participated in this study with 97% response rate. The prevalence of visual impairment was 37.58% (95% CI: 32.3, 42.9). Age (AOR: 1.06, 95% CI: 1.02, 1.09, *p* < or = 0.001), poor regular exercise (AOR = 2.91, 95%CI: 1.47–5.76, *p* < or = 0.001), duration of DM above 5 years (AOR = 2.42, 95% CI: 1.25–4.73, *p* < or = 0.01), insulin treatment (AOR = 14.05, 95% CI: 2.72, 72.35, *p* < or = 0.01), and poor glycemic control (AOR = 2.17, 95% CI: 1.13–4.14, *p* < 0.05) were statistically associated with visual impairment.

**Conclusion:**

The prevalence of visual impairment in Dessie town hospitals accounted for more than a third of patients living with T2DM. Visual impairment is associated with increased age, poor regular exercise, longer duration of DM, and insulin treatment. Thus, early detection of VI through screening and regular follow-up is recommended to reduce the risk of VI and vision loss.

## Background

Type 2 diabetes mellitus (T2DM) is a devastating metabolic disease, occurring in developed and developing countries [1]. Patients with T2DM are highly prone to cardiovascular diseases, renal failure, neurological problems, retinopathy, and visual impairment [2, 3]. Even though some forms of diabetic retinopathy are restorable, most problems of vision loss are not restorable [1–3]. People developing visual impairment(s) experience major life changes, such as general health limitations or loss of nearby family member [4]. Visual impairment (VI) is a functional limitation of the eye(s) due to different medical problems, including T2DM, that results in reduced visual acuity, visual field loss, visual distortion, perceptual difficulties, or any combination of the above [5]. It is presented visual acuity of worse than 20/40 to no light perception (NLP) in either or both eyes [6]. Visual impairment can be congenital or hereditary. Causes of VI incudes refractive error, cataract, glaucoma, corneal opacity, age-related macular degeneration, diabetic retinopathy [5, 7–10], ocular disease, trauma and systemic diseases such as hyperthyroidism [11], rheumatoid arthritis, HIV/AIDS, and hypertension [12].

Unless early preventions made, visual impairment can lead unemployment, reduced productivity, increases medical expense, difficulties in reading and fail to work in order to obtain wages to their family, and execute their social responsibilities as a whole [7, 13]. They also are at higher risk of violence and abuse which limit them from participating in and contributing to their families and societies on an equal basis with others [14].

Globally, prevalence of VI increased sharply from 441.1 million [15] to 2.2 billion [7]. Of which at least 1 billion of VI could have been prevented. Among the causes of visual impairment, 1% was associated with diabetic retinopathy [16].

Different studies revealed discrepant prevalence of VI among T2DM as observed in Sankara Nethralaya (4%) [17], Peru (26.3%) [18], China (10%) [9], Jordan (17.7%) [19], Turkey (16.2%) [20], and Yemen (76.5%) [21]. In Africa, studies on VI among T2DM showed that 78.25% in South Africa [22], 17.1% in Zambia [23], 18.4% in Ghana [24], 24.1% in Nigeria [25]. Moreover observational studies in Tunisia [26] and Cameron [27] revealed 22.2 and 22.6% prevalence of VI among DM patients, respectively.

Visual impairment is associated with increased age, longer duration of DM, higher body mass index, lower educational level, and use of insulin as treatment option [9, 19, 28]. In Sankara Nethralaya, patients aged above 60 years and low socio-economic status [17] whereas a study in Peru revealed the association of VI with hypertension, hemoglobin A1c, and use of insulin [18]. Studies in Cameroon, Zambia, Turkey and Tunisia indicated that increased age, overweight, longer duration of DM, poor glycemic control, high systolic blood pressure, and use of insulin as treatment option for DM were significantly associated to VI [20, 23, 26, 29].

Even though there is one study in Ethiopia that revealed 28.9% of visual disturbance (not visual impairment) [30], there was no study conducted regarding VI and its predictors. Therefore, this study aimed to determine the prevalence of VI and its predictors among people living with T2DM at Dessie Town Hospitals, Northeast Ethiopia, 2020.

## Methods

### Study design, setting and period

This institution-based cross-sectional study was conducted at Dessie town Hospitals (both government and private Hospitals) from 15 February to 15 March 2020. Dessie town is located 400 km away from Addis Ababa, the capital city of Ethiopia. There are four private and one governmental referral Hospitals in Dessie town. According to annual summative report of all diabetic clinics in Dessie town hospitals, an estimated number of 14,000 people living with T2DM were served. In each Hospital, there was diabetic follow-up clinic for people living with DM. The follow-up date in each hospital was from Monday to Friday.

### Study population and eligibility criteria

All people living with T2DM visiting diabetic clinic of each Hospital during data collection period were eligible for the study. People living with T2DM who were seriously ill, pregnant women and those who had HIV/AIDS, trachoma, acute eye infections and trauma to the eye and history of head injury, history of stroke were excluded from the study.

### Sample size determination and sampling technique

Sample size was determined using a single population proportion formula with assumptions; *p* = 0.289 [30] (proportion of T2DM with visual disturbance), 95% CI, and 5% margin of error (d = 0.05). The minimum sample size was 316 and the final sample size was 332 after adding 5% oversampling to account for unpredictable events during data collection.

Samples were recruited using simple random sampling technique and allocated proportionally to the four hospitals based on the number of T2DM clients at each hospital. Order of follow-up was the sampling frame for selecting study participants.

### Operational definitions

#### Visual impairment

**A**ny loss or abnormality in an anatomical structure or a physiological or psychological function [5]. It is presenting visual acuity of worse than either 20/40 to no light perception (NLP) in either or both eyes which includes both low vision and blindness [31].

#### Low vision

Even with corrective lenses, it is inability to clearly see at a distance of 6 m (20 ft) that individuals with normal vision can clearly see at a distance of 12 m (40 ft) or visual acuity between 6/12 and 3/60. Low vision includes mild visual impairment (VA between 6/12 and 6/18), ‘moderate visual impairment (VA between 6/18 and 6/60)’ and ‘severe visual impairment (VA between 6/60 and 3/60)’ from all causes [7].

#### Blindness

Inability to read the largest letter on a vision chart at a distance of 3 m (10 ft) or visual acuity was worse than 3/60 [32].

#### Visual acuity/VA

Simple, non-invasive measure of the visual system’s ability to discriminate two high contrast points in space. It is usually taken at a distance of 6 m or 20 ft [16]. It is expressed in terms of A/B, where A is the distance between observer and letters and B is the expected distance that the normal eye can see.

#### Obesity

Body Mass Index (BMI) of ≥30 kg/m^2^. Subtypes include class 1 obesity (BMI: 30–34.9 kg/m^2^) class 2 obesity (BMI:35–39.9 kg/m^2^) class 3 extreme obesity or morbid obesity (BMI: ≥40 kg/m^2^) [33]. On the other hand: BMI < 18.5 kg/m^2^ (underweight), 18.5–24.9 kg/m^2^ (normal) and 25–29.9 kg/m^2^ (overweight).

#### Exercise

A person who experienced regular exercise below 150 min (3-5 days) per week was considered as having *poor regular exercise* otherwise it was considered as having *good regular exercise* [34].

#### Glycemic control level

*Good* when recorded fast blood sugar (FBS) was below 152 mg/dl and *poor* when FBS was 152 mg/dl and above [35].

### Data collection instrument, procedure and quality control

Pre-tested, semi-structured-interviewer-administered questionnaire containing Socio-demographic variables, behavioral variables (diet, exercise and regular follow-up), clinical related variables (glycemic control, plasma glucose level, duration of DM, treatment option for DM, and presence of comorbidities such as hypertension and/or obesity) were used to collect data. Exit interview was taken and participants were scheduled for eye examination at the eye clinic of the same institution on the same day. The participants had gotten counseling, care and referral depending on the ocular findings**.** For the measurement section, we used **t**ape meter, and weight balance for height and weight, respectively. Each participant had visual acuity assessment with illuminated Snellen’s chart for each eye at 6 m. Training was given to data collectors and the supervisor by principal investigator about the objectives of the study, data collection techniques and ethical issues. Data collectors were four (2 BSc ophthalmic Nurses and 2 BSc Nurses) and one supervisor (BSc Public health). Strict supervision was taken during data collection process.

### Reliability and validity

The value of Cronbach’s alphas (α) in this study was < 0.5. This was because we used self-developed questionnaires and was unstandardized. We conduct a pre-test on 17 students out of the study area to ensure content validity. The tool was improved based on added findings from pre-test. Those questions having unclear meaning were rewritten for well understanding of study participants.

### Data processing and statistical analysis

Data were entered into Epi-Data 3.1 and exported into SPSS 22 for statistical analysis. Continues data were described by median and interquartile range (IQR) whereas frequency with percent was used to describe categorical variables. Bi-variable binary logistic regression analysis was performed to select potential candidate variables for the final model with cut-off point of *p*-value ≤0.25 [36]. Multivariable binary logistic regression analysis was executed to identify predictors of visual impairment. The measure of association was described by crude odds ratio (COR) and adjusted odds ratio (AOR) with their 95% CI. In the final model, variables with a *p*-value < 0.05 were considered as statistically significant. Model fitness was checked by Hosmer and Lemeshow goodness of fit test (at *p* > 0.05).

## Results

Three hundred and twenty-two people living with T2DM participated in the study yielding a response rate of 97%. The median age of participants was 52 years (IQR: 45–60 years). 54.3% of participants were males with female to male ratio of 1: 1.18. Seventy-three (22.7%) participants did not attend formal education. Majority of individuals 291 (90.4%) were married, and 249 (77.3%) were urban dwellers. The participants claim a median monthly income of 3570 Ethiopian birr (ETB) (IQR: 2000–5195, Min = 800 and Max = 9600 ETB) (Table 1).

**Table 1:** Socio-demographic characteristics of people living with T2DM at Dessie town Hospitals, Northeast Ethiopia, 2020 (*n* = 322)

Variables	Categories	Frequency	Percent
Age in years	20–40	37	11.5
	41–59	189	58.7
	60–87	96	29.8
Sex	Male	175	54.3
	Female	147	45.7
Religion	Muslim	212	65.8
	Orthodox	103	32
	Others^a^	7	2.2
Marital status	Never married	31	9.6
	Married	291	90.4
Residence	Urban	249	77.3
	Rural	73	22.7
Educational level	No formal education	73	22.7
	Primary	84	26.1
	Secondary	79	24.5
	Diploma and above	86	26.7
Occupation	Government workers	70	21.8
	Private workers ^b^	115	35.7
	Farmer	37	11.5
	House wife	60	18.6
	Others^c^	40	12.4

Other^a^: protestant and catholic, Private worker^b^: construction, daily laborer, driver, mechanic, merchant, others^c^: jobless, pensioner

### Vision related characteristics of participants

In this study, one hundred and seventy-three (53.7%) participants experienced a duration of 5 years and lower since diagnosis whereas 24(7.5%) were new cases. Two hundred and sixty-four (82.0%) had regular follow-up for diabetes the hospitals. One hundred and ninety-nine (61.8%) participants had poor regular physical exercise. The treatment option for DM was oral hypoglycemic agents (62.4%). One-third of diabetes 98 (30.4%) had co-morbid hypertension and 187 (58.1%) of them had poor glycemic control (Table 2).

**Table 2:** Vision-related characteristics of participants at Dessie town Hospitals, Northeast Ethiopia, 2020 (*n* = 322)

Variables	Categories	Frequency	Percent
Have trouble in adjusting light (night blindness)	Yes	46	14.3
	No	276	85.7
Regular follow-up	Yes	264	82
	No	58	18
Duration of diabetes (years)	Newly diagnosed	24	7.5
	≤5	173	53.7
	> 5 up to 24	125	25.8
Treatment (*n* = 296)	OHA without insulin	201	67.9
	Both of OHA and insulin	73	24.7
	Insulin only	22	7.4
Regular exercise	Good*	123	38.2
	Poor*	199	61.8
BMI (kg/m^2^)	Underweight	5	1.5
	Normal	159	49.4
	Overweight	121	37.6
	Obese	37	11.5
Comorbid hypertension	Yes	98	30.4
	No	224	69.6
Glycemic control	Good	135	41.9
	Poor	187	58.1

Note: *OHA* Oral Hypoglycemic Agent

### Prevalence of visual impairment among people living with T2DM

In the current study, the prevalence of visual impairment was 37.58% [95% CI: 32.3–42.9%]. Among the overall prevalence of visual impairment, 43(35.5%) had bilateral vision impairment and 78 (64.5%) had monocular vision impairment. Of all visually impaired T2DM, 107 (88.4%) and 14 (11.6%) had low vision and blindness, respectively (Table 3).

**Table 3:** Forms of visual impairment categories among people living with T2DM at Dessie town Hospitals, Northeast Ethiopia, 2020, (*n* = 322)

Visual impairment category		Frequency	Percent
< 6/12–6/18	Bilateral mild VI	28	23.1
< 6/18–6/60	Bilateral moderate VI	10	8.3
< 6/60–3/60	Bilateral severe VI	1	0.8
< 3/60-NLP	Bilateral blindness	4	3.3
< 6/12–6/18, other eye 6/6–6/12	Monocular mild VI	0	0.0
< 6/18–6/60, other eye 6/6–6/18	Monocular moderate VI	48	39.7
< 6/60–3/60, other eye 6/6–6/60	Monocular severe VI	20	16.5
< 3/60-NLP, other eye 6/6–3/60	Monocular blindness	10	8.3
Total		121	100

Note: *NLP* no light perception, *VI* visual impairment

### Predictors of VI among people living with T2DM

Age, sex, marital status, educational level, occupation, residence, regular physical exercise, duration of DM, treatment option for DM, baseline random plasma glucose, co-morbid hypertension, and glycemic control were independently associated with VI in binary logistic regression. After running the aforementioned variables in the multivariable binary logistic regression; increased age, poor regular physical exercise, longer duration of T2DM, use of insulin as treatment option for DM and, poor glycemic control were statistically associated with VI.

The odds of having VI for each age increase of a unit was 1.06 times (AOR: 1.06, 95% CI: 1.02, 1.09). Participants who relied on insulin as treatment option for DM were 14 times (AOR = 14.05, 95% CI: 2.72, 72.35) more likely to get VI than those who used treatment options without insulin. The odds of having VI was 2.91 times (AOR = 2.91, 95% CI: 1.47, 5.76) higher among those who had poor physical exercise than those who were good in regular physical exercise. People living with T2DM for a duration of more than 5 years were 2.42 times (AOR: 2.42, 95% CI: 1.24, 4.73) more likely to acquire VI than those with duration of 5 years and below. Those who had poor glycemic control were 2.17 times (AOR: 2.17, 95% CI: 1.13, 4.14) more likely to develop VI as compared to those who had good glycemic control (Table 4).

**Table 4:** Multivariable binary logistic regression analysis for predictors of visual impairment among people living with T2DM at Dessie town Hospitals, Northeast Ethiopia, 2020 (*n* = 322)

Variables	Categories	VI		COR (95% CI)	AOR(95% CI)
		No	Yes		
Sex	Male	119	56	1	1
	Female	82	65	1.68 (1.06, 2.65)	1.29(0.56, 2.96)
Marital status	Unmarried	27	4	1	
	Married	174	117	4.54(1.54, 13.31)	1.25(0.30, 5.11)
Education	Unable to read and write	32	41	1	1
	Primary	52	32	0.48 (0.25, 0.90)	0.92 (0.33 2.50)
	Secondary	51	28	0.43 (0.22, 0.82)	1.11 (0.37, 3.29)
	Diploma and above	66	20	0.23 (0.12, 0.46)	0.67 (0.20, 2.23)
Occupation	Government worker	52	18	1	1
	House wife	26	34	3.77 (1.80, 7.92)	1.54 (0.59, 4.00)
	Other	19	21	3.19 (1.40, 7.25)	1.33(0.42, 4.17)
Residence	Urban	166	83	1	
	Rural	35	38	2.17(1.27, 3.68)	1.85(0.71, 4.80)
Regular-exercise	Good	94	29	1	
	Poor	107	92	2.78(1.68, 4.59)	2.91(1.47, 5.76)**
Duration of diabetes	≤5 years	154	43	1	
	> 5–24 years	47	78	28.59(3.68, 221.75)	2.42(1.24, 4.73)**
Treatment	OHA without insulin	140	61	1	
	Both of OHA and insulin	34	39	2.63(1.52, 4.56)	1.45(0.71, 2.97)
	Insulin only	2	20	22.95(5.20, 101.25)	14.05(2.72, 72.35)**
Hypertension	Yes	51	47	1.86(1.15, 3.03)	1.26(0.65, 2.44)
	No	150	74	1	
Glycemic Control (FPG)	< 152 (good)	94	41	1	
	≥152 (poor)	107	80	1.71(1.07, 2.73)	2.17(1.13, 4.14)*

*OHA* oral hypoglycemic agent, *significant at *p* < 0.05, **significant at *p* < 0.01, ***significant at *p* < 0.001, 1 = indicator/reference category, Hosmer-Lemeshow goodness-of-fit (*p* = 0.781), no multicollinearity (VIF < 10)

## Discussion

In this institution based cross sectional study, VI among T2DM patients and its predictors has been assessed and it was found that 37.58% of T2DM patients were visually impaired. Participants with older age, poor physical activity, longer diabetes duration and poor glycemic control were significantly associated factors for VI.

The prevalence of visual impairment in the current study is 37.58% (95% CI: 32.3–42.9) which is higher than previous study at the same study area (i.e. Dessie) which was 28.9% [30]. This difference is probably due to the type of study design used by previous researchers such as review of patient records as source of data, study population difference (only newly diagnosed DM) and visual disturbance was detected by clinical findings and questionnaire based approaches (where visual acuity test was not applied for the former study). Also, unlike to this study, the previous study conducted only at a single hospital.

Furthermore, the prevalence of visual impairment in the current study is higher than other studies in Nigeria (24.1%) [37], Tunisia (22.2%) [26], Cameron (29.7%) [29], Ghana (18.4%) [24], Zambia (17.1%) [23], Turkey (13.5%) [20], Peru (40.2%) [18], Jordan (17.7%) [19], Southern China (10%) [31], and Sankara Nethralaya (4.1%) [17]. This discrepancy is most likely due to differences in the case definition, socio-economic, and quality of chronic disease care service. In our study, the cut-off point was VA < 6/12 to define VI while VA < 6/18 was used to define VI for the above listed studies. Those studies used the better eye presenting visual acuity to define visual impairment unlike the present study. Moreover the other studies were conducted at the community level where chance of screening normal sighted people existed. In contrary to this, our study was hospital-based where most patients came with noted diabetic complication which may lead to increased prevalence of VI. Nigerian study applied volunteer sampling and smaller sample size. In Tunisia, purposive sampling was carried out which might introduce bias and both studies used the better eye presenting visual acuity to define VI. If one eye was visually impaired and the other was not impaired, they considered as no VI which could underestimate the magnitude of VI compared to our study which is done by considering either eye’s visual acuity.

The prevalence of unilateral visual impairment in this study is lower than studies done in Yemen (76.5% [21] and south Africa (78.25%) [22]. This variation might be due to differences in the case definition of VI and sample size. Study participants were T2DM with an age of 40 years and above with a cut-off point of VA between 6/9.5 and 6/18 which was defined as a VI for the study conducted in South Africa while the large sample size was used in Yemen with all conditions overestimate or had possibilities to add additional visually impaired cases.

In the current study increased age, poor regular exercise, longer duration of T2DM, use of insulin as treatment option, and poor glycemic control were predictors (statistically significant) of VI. The odd of having VI for each age increase of a unit was 1.06 times. This is similar to a study in Tunisia [26], Southern China [9], and Sankara Nethralaya [17]. Possible reasons for this might be with advanced age there might be decreased in physical exercise, loss of muscle mass, and gain weight that in turn fatty cells had more resistant for insulin that increase hyperglycemia. Furthermore, advanced age has potential risk of macrovascular events due to cardiac insufficiency [38].

The odds of having VI among people living with T2DM who had poor physical exercise was 2.91 times more likely than those who reported good physical exercise. This might be due to exercise can promote an increase in the bioavailability of nitric oxide which decrease blood pressure, post exercise can increase glycolipid uptake and utilization which improves glucose homeostasis, insulin sensitivity, and maintaining glycemic level [39–41], optimized body mass index and modulated DNA methylation [42].

Participants with duration of diabetes of above 5 years was 2.42 times more likely to get visual impairment as compared to those with type 2 diabetes duration of 5 years and below. This finding is in line with Zambia [23], Yemen [28], Peru [18], and China [9]. The possible reason might be longer duration of T2DM might reduce adherence to self-care [43], hall marker for long-term exposure to hyperglycemia [44] and potentially increases risk of macrovascular and microvascular events and death [38]. Moreover, longer duration is linked to a reduction in insulin secretion or excessive insulin resistance in people living with T2DM [45].

People living with T2DM who relied on insulin only as treatment option were 14 times more likely to get VI than those who used oral hypoglycemic agents without insulin. This is consistence with studies in Zambia [23], Turkey [20], Peru [18], Jordan [19], and Sankara Nethralaya [28]. The reason is probably linked to the use of insulin alone that reflects less adherence [43] resulting in deterioration in kidney function, decline β-cell function or increase insulin resistance over time [46] which is associated with poor plasma glucose control and higher risk of severe diabetes.

The odds of being visually impaired was 2 times higher among participants with poor glycemic control in contrary to good glycemic control which is in line with study in Peruvian [18]. The possible reason might be poor glycemic control or persistent hyperglycemia damages retinal vasculature via activation of pro-inflammatory mediators such as tumor necrotic factor (TNF)-2, interleukin-6, interleukin-1b, angiotensin II, endothelin-1, and vascular endothelial growth factor (VEGF) that could alter retinal blood barrier leads to retinal vessel leakage causing macular edema and nerve scaring which result in retinal detachment and sudden vision loss.

### Limitations of the study

Since the study was cross sectional, it could not show cause-effect relationship. Recall and social desirability biases were also be possible limitations. Categorization of VA was based on presenting, not corrected visual acuity. HbA1c was not measured due to clients’ financial issue so that physicians ordered fast plasma glucose instead of HbA1c.

## Conclusion

The prevalence of VI in Dessie town hospitals accounts for more than a third of patients living with T2DM that implied a significant public health problem. Older age, poor regular physical exercise, longer duration of T2DM, use of insulin as treatment option for DM, and poor glycemic control were predictors of VI among people living with T2DM. Regular diabetes follow-up and visual screening for all people living with T2DM should be done at older age group patients and for those having longer duration of DM which can reduce visual morbidity and vision loss. Patients should control glycemic level by taking medications and through adequate and regular physical exercise. Public health policies with educational programs and promotion of DR screening of all T2DM are needed and timely management of DR that greatly reduce the incidence of visual impairment due to diabetes.

## Data Availability

The dataset of this research is available at the corresponding author and can be obtained with reasonable request.

## Abbreviations

AOR: Adjusted Odds Ratio
BMI: Body Mass Index
CI: Confidence Interval
DM: Diabetes Mellitus
DR: Diabetic retinopathy
FPG: Fasting Plasma Glucose
HIV/AIDS: Human Immune Virus/Acquired Immune Deficiency Syndrome
IQR: Inter Quartile Range
NLP: No Light Perception
RPG: Random Plasma Glucose
SPSS: Statistical Package for Social Sciences
SRS: Simple random sampling
T2DM: Type 2 diabetes mellitus
VA: Visual acuity
VI: Visual impairment
